# Elephant Endotheliotropic Herpesvirus Hemorrhagic Disease in Asian Elephant Calves in Logging Camps, Myanmar

**DOI:** 10.3201/eid2601.190159

**Published:** 2020-01

**Authors:** Zaw Min Oo, Ye Htut Aung, Tin Tun Aung, Nyo San, Zaw Min Tun, Gary S. Hayward, Arun Zachariah

**Affiliations:** University of Veterinary Sciences, Yezin, Myanmar (Z.M. Oo, Y.H. Aung);; Ministry of Environmental Conservation and Forestry, Yangon, Myanmar (Z.M. Oo, T.T. Aung, N. San, Z.M. Tun);; Johns Hopkins School of Medicine, Baltimore, Maryland, USA (G.S. Hayward);; Kerala Veterinary and Animal Sciences University, Wayanad, India (A. Zachariah)

**Keywords:** elephant endotheliotropic herpesvirus, EEHV, viruses, elephant endotheliotropic herpesvirus hemorrhagic disease, elephants, Asian elephant calves, logging camps, endangered species, hemorrhagic disease, virus subtypes, hypervariable genes, Myanmar

## Abstract

In recent years, an alarming number of cases of lethal acute hemorrhagic disease have occurred in Asian elephant calves raised in logging camps in Myanmar. To determine whether these deaths were associated with infection by elephant endotheliotropic herpesvirus (EEHV), we conducted diagnostic PCR subtype DNA sequencing analysis on necropsy tissue samples collected from 3 locations. We found that EEHV DNA from 7 PCR loci was present at high levels in all 3 calves and was the same EEHV1A virus type that has been described in North America, Europe, and other parts of Asia. However, when analyzed over 5,610 bp, the strains showed major differences from each other and from all previously characterized EEHV1A strains. We conclude that these 3 elephant calves in Myanmar died from the same herpesvirus disease that has afflicted young Asian elephants in other countries over the past 20 years.

In 1990, a brief description of a case of lethal acute hemorrhagic disease in a young Asian circus elephant in Switzerland that apparently involved a previously unknown herpesvirus was reported by Ossent et al. (*1*). However, it was not until the study of Richman et al. (*2*,*3*), published in 1999, that this disease was shown to be associated with a novel herpesvirus designated elephant endotheliotropic herpesvirus (EEHV) 1 because of detection of nuclear inclusion bodies in damaged vascular endothelial cells in diseased heart and liver tissues. Those 2 studies provided evidence for the presence of high levels of small segments of the DNA genome of EEHV1 found by PCR sequencing techniques in blood samples and necropsy tissue samples from all major internal organs during active cases of hemorrhagic disease in 5 Asian elephant calves in zoos in North America and Europe, as well as in archival tissue block samples from 6 other cases in older elephants. Only 2 of the afflicted calves with less severe signs survived the acute disease after treatment with the human antiherpesvirus drug famciclovir; and viral DNA load in their blood was documented to decrease below levels of detection over a 4–6-week convalescence and recovery period. One young African elephant calf from a zoo that had a similar lethal hemorrhagic disease case had DNA from a second related virus species (EEHV2).

Since these original reports, numerous studies reviewed by Hayward (*4*) and Long et al. (*5*) have shown the presence of either of 2 chimeric subtypes of EEHV1A or EEHV1B in <50 additional lethal cases and 10 drug-treated confirmed DNA-positive survivors with signs of disease in mostly young Asian elephants in Europe and North America (*6*–*8*). A small number of lethal and nonlethal cases of viral DNA–positive hemorrhagic disease cases in young Asian elephants involved 2 additional related but considerably diverged species of *Proboscivirus* (EEHV4 and EEHV5) (*9*–*16*). EEHV4 is estimated to have last had common ancestors with EEHV1 35 million years ago and EEHV5 is estimated to have last had common ancestors with EEHV1 20 million years ago.

Furthermore, many examples of occasional shedding of these viruses, especially in trunk-wash and saliva samples have also been documented from adult Asian and African elephants with signs of disease (*17*–*20*; V.R. Pearson et al., Fox Chase Cancer Center, pers. comm., 2019 Jan 22). All currently available data (*5*,*13*) support the concept that EEHV1A, EEHV1B, EEHV4, and EEHV5 are endemic in Asian elephants (*Elephas maximus*), whereas EEHV2, EEHV3, EEHV6, and EEHV7 are endemic to and largely ubiquitous in African elephants (*Loxodonta africana*). A subset of ≈20% of immunologically naive young Asian elephants 1–8 years of age are susceptible to the most severe forms of hemorrhagic disease that are caused predominantly by primary infections with the most pathogenic version of these viruses (EEHV1A).

A major aspect of the situation concerning possible long-term effects on breeding and survival of critically endangered Asian elephants worldwide is whether these viruses and the same hemorrhagic disease occur within countries in Asia and especially in wild populations. Reid et al. (*21*) reported an EEHV1 DNA–positive case from Cambodia. However, it was not until Zachariah et al. (*22*) published their results, after setting up a diagnostic PCR DNA laboratory in southern India to examine necropsy tissue collected from young orphan and wild elephants that suddenly died, that the range of the disease and virus became firmly established. These authors initially described 8 lethal cases of hemorrhagic disease associated with EEHV1A and 1 case associated with EEHV1B from India. Later in the same year, Sripiboon et al. (*23*) also reported 2 cases involving EEHV1A and EEHV4 from Thailand. More recent studies have also confirmed 2 lethal cases of EEHV1 hemorrhagic disease in Laos (*24*) and as many as 15 additional cases in Thailand (*25*,*26*).

We have also expanded the studies in India to 22 lethal cases, including 12 in wild free-ranging calves (*27*). In this study, we report DNA sequence–confirmed cases of lethal EEHV1A disease in 3 young logging camp elephants in Myanmar, including partial genetic analysis by PCR sequencing of the strains involved and comparison with all the cases from India and other representative cases worldwide.

## Materials and Methods

Case 1 (M1) was in a 20-month-old, captive-born, female *E. maximus* calf that died suddenly on February 11, 2012, in Minbu District, Magway region, Myanmar. Case 2 (M2) was in a 22-month-old, captive-born, male *E. maximus* calf that died suddenly on September 14, 2013, in Nyaung Lay Pin District, Bago (East) region, Myanmar. Case 3 (M3) was in a 16-month-old, captive-born, female *E. maximus* calf that died suddenly on January 8, 2014, in Nay Pyi Taw District, Myanmar.

We observed typical multiorgan hemorrhages, particularly in heart, liver, and peritoneum. We collected tissue samples and placed them in molecular-grade absolute ethanol during postmortem and stored them at −20°C. We used liver samples from each case for extraction of intracellular DNA by using the QIAamp Blood and Tissue Mini Kit (QIAGEN, https://www.qiagen.com). Conventional PCR amplifications were performed at the University of Veterinary Sciences (Yezin, Myanmar) during an EEHV workshop. These DNA samples were used as templates to amplify and sequence 7 standard preferred EEHV1 PCR DNA loci (*22*,*27*) from all 3 cases, as well as an eighth locus (U51, vGPCR1) from 1 of these cases. We performed Sanger DNA sequencing and DNA sequence editing and constructed phylogenetic trees as described (*6*,*10*,*22*). We used the updated sets of PCR primers reported by Zachariah et al. (*27*).

We generated detailed results for genetic differences between EEHV DNA samples at each PCR locus as linear nucleotide-level polymorphisms by using Geneious software (https://www.geneious.com) from alignments made in Muscle software (https://www.drive5.com/muscle) or as Bayesian nearest neighbor–based phylogenetic trees at the DNA or protein level. For comparative purposes, we combined results for Myanmar (MP#) with all data for samples from India (IP#) and Sumatra (SP#) evaluated by our group. We also showed levels of divergence from prototype samples from North America (NAP#) or Europe (EP#) for each known subtype. We used other EEHV reference strains, such as EEHV1B, EEHV2, EEHV5, or EEHV6, as outgroups.

## Results

**Figure 1:**
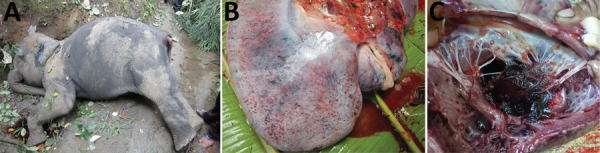
Gross morphologic characteristics of Asian elephant calf no. 3 (MP03) that had endotheliotropic herpesvirus hemorrhagic disease in logging camp, Myanmar. A) Before postmortem necropsy. B) Hemorrhagic lesions on intact surface of the liver. C) Hemorrhagic lesions inside the atrium and valves of the dissected heart.

We evaluated 3 cases of fatal hemorrhagic disease in captive-born *E. maximus* calves reared in camps in Myanmar. We provide gross morphology for 1 of the cases (Figure 1). The viral genomes in necropsy tissue DNA samples were designated MP01, MP02, and MP03 (for Myanmar *Proboscivirus* case numbers). Overall results of gene subtyping PCR analyses (Table 1) and assigned GenBank accession numbers (Table 2) are provided.

**Table 1:** EEHV PCR gene locus subtype data for cases of hemorrhagic disease in 3 Asian elephant calves, Myanmar*

Case	PCR gene locus							
	E5	vGPCR1	U71-gM	POL-AB	TER	gH-TK	HEL	vOX2
MP01, Myanmar1	B1	ND	A4	A	A1	D	A1	1
MP02, Myanmar2	B1	ND	A4	A	C	D	C2	2
MP03, Myanmar3	C1	E	A5	A	A3	D	A2	3

*EEHV, elephant endotheliotropic herpesvirus; ND, not determined; 1, identical to IP143 from India; 2, novel variant not seen previously; 3, identical to IP93 from India.

**Table 2:** GenBank DNA file accession numbers for cases of hemorrhagic disease in 3 Asian elephant calves, Myanmar*

Case, virus code	Size, bp	MP01	MP02	MP03
E3(vGPCR5)	962	MF579041	MF579042	MF577043
U38(POL)	485	MF579060	MF579061	MF579062
U48(gH-TK)	850	MF464877	MF464878	MF464879
U51(vGPCR1)	677	ND	ND	MF579097
U60(TERex3) L	741/724/726	MF579075	MF579076	MF579077
U71-gM	651	MF579110	MF579111	MF579112
U77(HEL)	952/921/952	MF579126	MF579127	MF579128
E54(vOX2-1)	854	MF464888	MF464889	MF464890

*ND, not done.

At the time this study was conducted, we selected PCR primers provided to participants at a workshop in Myanmar after extensive previous analysis of 10 cases from Europe and 26 cases from North America as preferred standard sets that amplify 8 gene loci distributed across the 180–206-kb genomes of EEHV viruses. These gene loci included 4 well-conserved gene loci, U38(POL), U60(TERex3), U71/gM, and U77(HEL), which enabled identification of specific EEHV viruses present, as well as resolved chimeric EEHV1A subtype versus EEHV1B subtype strains (*6*). In addition, we used 4 other PCR loci that encompass parts of well-characterized hypervariable genes. These loci were E5(vGPCR5), U48(gH-TK), U51(vGPCR1), and E54(vOX2-1); we used them to address levels and patterns of individual variability for comparison with known, highly divergent, worldwide populations of EEHV1 strains.

**Figure 2:**
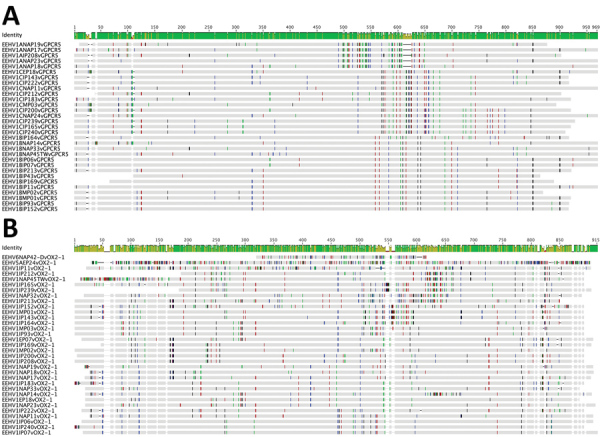
Nucleotide sequence polymorphism charts for Asian elephant calves that had endotheliotropic herpesvirus hemorrhagic disease in logging camp, Myanmar. Shown are comparisons for hemorrhagic disease cases MP01, MP02, and MP03 across 2 hypervariable EEHV1 PCR loci. A) E5(vGPCR5). B) E54(vOX2-1). Polymorphisms were generated by using Geneious (https://www.geneious.com) and MEGA5 (https://www.megasoftware.net) Bayesian phylogenetic trees comparing the Myanmar *Proboscivirus* case DNA sequence results with matching data available for all 22 cases from India (IP#) and 2 cases from Sumatra (SP#) plus several representative cases from North America (NAP#) and Europe (EP#) available in GenBank. Both prototype EEHV2(NAP12, Kijana) and EEHV5A(EP24, Vijay) genomes shown in the top lines were used as references in panel B, and EEHV1B(NAP19) was used as the reference for panel A. Assigned subtypes are included with designated code numbers listed on the left-hand side for each genome. Colored short vertical lines indicate single-nucleotide differences from the consensus sequences of all the genomes shown in each panel. Gaps or missing sequence segments appear as blank spaces. EEHV, elephant endotheliotropic herpesvirus.

**Figure 3:**
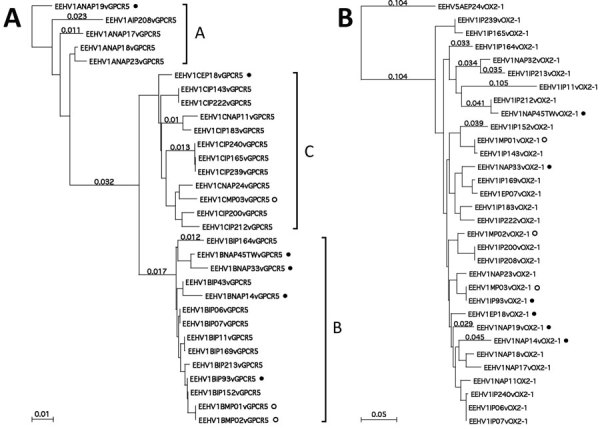
Protein level phylogenetic trees for Asian elephant calves that had endotheliotropic herpesvirus hemorrhagic disease in logging camp, Myanmar. Shown are comparison of examples of Asian EEHV1 at 2 representative hypervariable loci. A) EEHV1 E5(vGPCR5). B) E54(vOX2-1). Bayesian linear phylogenetic trees were generated from translated amino acid data in MEGA5.4.6 (https://www.megasoftware.net) by using similar aligned datasets as in Figure 2. Evolutionary history was inferred by using the maximum-likelihood method based on the Jones–Taylor–Thornton matrix-based model with the bootstrap consensus tree. Analysis in panel A involved 31 nt sequences with 303 aa positions in the final dataset with EEHV1B(NAP19) as outgroup. Analysis in panel B involved 32 nt sequences with 244 aa positions in the final dataset and with EEHV5A(EP24) as outgroup. Numbers along branches are bootstrap values. Some representative branch length values are provided. The 3 major subtype clusters are indicated as A, B, or C for E5(vGPCR5) along the right side of panel A, but no similar dramatic subtype clustering was discernable for E54(vOX2-1). All 6 examples in which the genomes have classic EEHV1B type core chimeric domain (CDI, CDII, and CDIII) features elsewhere are indicated by solid circles. Positions of the 3 cases from Myanmar are indicated by open circles. Scale bars indicate amino acid substitutions per site. EEHV, elephant endotheliotropic herpesvirus.

We found that all 3 strains from Myanmar were EEHV1A variants that amplified strongly after the first-round conventional PCR. We identified nucleotide-level differences for 2 selected representative hypervariable PCR loci, namely E5(vGPCR5) and E54(vOX2-1) (Figure 2). Equivalent data for the U48(gH-TK) locus have already been reported for cases from India by Zachariah et al. (*27*). We provide 2 examples of the protein-level phylogenetic trees, again those for the E5(vGPCR5) and E54(vOX2-1) loci (Figure 3). The equivalent protein-level phylogenetic trees for U48(gH-TK) has been included in the report by Zachariah et al. (*27*). In addition, matching comparative DNA-level phylogenetic trees for all these samples at the 7 PCR loci other than U48(gH-TK) have been reported by Zachariah et al. (*27*). Finally, we report simple numerical difference comparisons (number and percentage) for the 3 strains from Myanmar with the prototype EEHV1A(NAP23, Kimba) and EEHV1B(EP18, Emelia) strains at the 3 most hypervariable loci, namely E5(vGPCR5), U48(gH-TK), and E54(vOX2-1) (Tables 3,4,5).

**Table 3:** Nucleotide differences between EEHV strains from Asian elephant calves in Myanmar and EEHV1A and EEHV1B prototypes at the E5(vGPCR5) locus*

vGPCR5	MP01	MP02	MP03	NAP23	EP18
MP01	–	0	6.9	7.6	5.6
MP02	0	–	6.9	7.6	5.6
MP03	61	61	–	9.3	2.5
NAP23	67	66	83	–	7.9
EP18	50	50	22	71	–

*Values to the right and above the dashes are percentages, and values to the left and below the dashes are number of nucleotides. EEHV, elephant endotheliotropic herpesvirus; EP18, prototype chimeric EEHV1B; NAP23, prototype EEHV1A.

**Table 4:** Nucleotide differences between EEHV strains from Asian elephant calves in Myanmar and EEHV1A and EEHV1B prototypes at the U48(gH-TK) locus*

gH-TK	MP01	MP02	MP03	NAP23	EP18
MP01	–	0	1.0	8.7	28
MP02	0	–	1.0	8.7	28
MP03	8	8	–	8.5	28
NAP23	73	73	71	–	28
EP18†	227	227	235	233	–

*Values to the right and above the dashes are percentages, and values to the left and below the dashes are number of nucleotides. EEHV, elephant endotheliotropic herpesvirus; EP18, prototype chimeric EEHV1B; NAP23, prototype EEHV1A.

**Table 5:** Nucleotide differences between EEHV strains from Asian elephant calves in Myanmar and EEHV1A and EEHV1B prototypes at the E54(vOX2-1) locus*

vOX-1	MP01	MP02	MP03	NAP23	EP18
MP01	–	9.7	8.7	10.1	10.2
MP02	82	–	2.8	6.1	5.7
MP03	74	28	–	3.7	5.0
NAP23	86	51	31	–	2.4
EP18	87	48	42	21	–

*Values to the right and above the dashes are percentages, and values to the left and below the dashes are number of nucleotides. EEHV, elephant endotheliotropic herpesvirus; EP18, prototype chimeric EEHV1B; NAP23, prototype EEHV1A.

Despite the consistent nucleotide divergence subtyping patterns, the 4 conserved loci evaluated rarely showed large numbers of amino acid polymorphisms. Even within the other 4 hypervariable loci, most nucleotide changes were also synonymous. In addition, unlike conserved loci, there are also numerous nonsynonymous amino acid changes, which provide additional robust supportive evidence for the same subtyping patterns being recognized in the trees at the DNA and protein levels. This finding is especially valid for the U48(gH) protein, which is the only PCR segment evaluated here that is entirely encompassed within 1 of the EEHV1B chimeric domains (CD-II). The fact that the MP01 and MP02 versions of E5(vGPCR5) protein sequences are identical is a relatively rare exception, but otherwise the divergence of most EEHV1 strains from each other is evident in E5(vGPCR5) and E54(vOX2-1) protein phylogenetic trees (Figure 2), and similar but different gene-specific patterns were also found in U48(gH-TK) and U51(vGPCR1) protein trees.

The 3 cases from Myanmar were caused by distinct strains that showed major differences from each other, as well as from other cases in Asia, North America, and Europe that we evaluated. However, there were unusual similarities among these 3 cases. For example, MP01 and MP02 were in the same subtype groupings at 4 loci, including 2 conserved loci, namely U71-gM and U38(POL), as well as 2 hypervariable loci, namely E5(vGPCR5) and U48(gH)-TK, but showed different subtypes for the other 3 loci, namely U60(TERex3), U77(HEL), and E54(vOX2-1), whereas MP03 was distinguishable from MP01 and MP02 at all loci except U38(POL). Similar to many of the cases from India, MP03 was a subtype E in U51(vGPCR1) and had 28 differences (4.1%) from the subtype A prototype EEHV1A(Kimba). MP01 and MP02 also had different subtypes (B1 for MP01 and C1 for MP02) from MP03 for E5(vGPCR5). Although all 3 cases from Myanmar had the same D subtype group within the U48(gH-TK) locus, MP03 still differed at this locus from the other 2 cases by 8 bp.

Although the captured E54(vOX2-1) gene is unusually well conserved when compared with the original host (African bush elephant [*Loxodonta africana*]) version at the protein level, it shows the greatest DNA-level divergence between all 3 strains from Myanmar and most other strains, in which MP01 differed from MP02 by 82 bp (9.7%), MP02 from MP03 by 28 bp (3.3%), and MP01 from MP03 by 74 bp (8.7%). Nevertheless, MP01 and MP03 were identical at nt positions 280–480, and MP02 and MP03 differed by only 1 bp over >50% of the locus between nt positions 364–851.

We found that MP01 and MP03 were identical to another case from India (MP01 with IP143 and MP03 with IP93) at the otherwise hypervariable E54(vOX2-1) locus, whereas MP02 had unique features different from all other cases that we have evaluated worldwide. Overall, across all 4 core conserved loci, U38(POL), U71-gM, U60(TERex3), and U77(HEL), the differences from the prototype EEHV1A(Kimba) strain were no more than 15 nt for MP01, 24 nt for MP02, and 22 nt for MP03. In contrast, the 3 most hypervariable loci showed a dramatically different pattern. In E5(vGPCR5), MP01 differed from Kimba by 67 (7.6%), MP02 by 66 (7.7%), and MP03by 83 (9.3%) nt. In U48(gH-TK), MP01 differed from Kimba by 73 (8.7%), MP02 by 73 (8.7%), and MP03 by 71 (8.5%) nt. Finally, in E54(vOX2-1), MP01 differed from Kimba by 86 (10.1%), MP02 by 66 51 (6.1%), and MP03 by 31 (3.7%) nt.

## Discussion

The DNA sequence and subtyping results for the hemorrhagic disease cases in Myanmar showed that they involved different independent EEHV1A strains that were distinct from all other strains examined in countries in Asia or in zoos in Europe and North America. A similar pattern of variability was also obtained for 21 of the 22 cases in India, but this pattern was not always found. In contrast, in every situation observed in which 2 cases of hemorrhagic disease occurred at the same facility at nearly the same time (i.e., within days or weeks of each other), genomes were always identical at all loci tested. These results include paired cases at 2 facilities in Asia (India and Sumatra), 2 cases in Europe (United Kingdom and Germany), and >3 cases in the United States (Texas, Florida, and Missouri). However, identical strains have not been reported for cases at different facilities (and different times), even in the same country. The fact that the 3 cases in Myanmar, although they showed some clear similarities across parts of the genomes and originated from within nearby geographic areas over a relatively short time frame, represent 3 distinct EEHV1A strains is not unexpected. However, more data from additional cases will be required to address whether overall populations of this virus in Myanmar have any common evolutionary features that differentiate them from the numerous and rather broadly diverged examples examined from India or Thailand.

Myanmar is now the sixth country in Asia (after Cambodia, Thailand, India, Laos, and Indonesia) in which apparent EEHV-associated hemorrhagic disease based on gross clinical or pathologic signs involving tissue hemorrhaging has been confirmed by PCR DNA subtype sequencing analysis. A brief preliminary speculation about these and multiple additional potential cases of similar hemorrhagic disease cases in Myanmar logging camps has been reported (*28*). Our findings will help with programs designed to address the increasing number of cases of lethal acute hemorrhagic disease in Asian elephants and possible long-term effects on breeding and survival of this critically endangered species.
